# Notch signaling and EMT in non-small cell lung cancer: biological significance and therapeutic application

**DOI:** 10.1186/s13045-014-0087-z

**Published:** 2014-12-05

**Authors:** Xun Yuan, Hua Wu, Na Han, Hanxiao Xu, Qian Chu, Shiying Yu, Yuan Chen, Kongming Wu

**Affiliations:** Department of Oncology, Tongji Hospital of Tongji Medical College, Huazhong University of Science and Technology, Building 303, 1095 Jie Fang Avenue, Wuhan, 430030 P.R. China

**Keywords:** Notch signaling, Epithelial-mesenchymal transition (EMT), Cancer stem cells (CSCs), Non-small-cell lung cancer (NSCLC), Gamma secretase inhibitor

## Abstract

Through epithelial-mesenchymal transition (EMT), cancer cells acquire enhanced ability of migration and invasion, stem cell like characteristics and therapeutic resistance. Notch signaling regulates cell-cell connection, cell polarity and motility during organ development. Recent studies demonstrate that Notch signaling plays an important role in lung cancer initiation and cross-talks with several transcriptional factors to enhance EMT, contributing to the progression of non-small cell lung cancer (NSCLC). Correspondingly, blocking of Notch signaling inhibits NSCLC migration and tumor growth by reversing EMT. Clinical trials have showed promising effect in some cancer patients received treatment with Notch1 inhibitor. This review attempts to provide an overview of the Notch signal in NSCLC: its biological significance and therapeutic application.

**Electronic supplementary material:**

The online version of this article (doi:10.1186/s13045-014-0087-z) contains supplementary material, which is available to authorized users.

## Current status of NSCLC

Lung cancer is a serious public health problem that affects the lives of millions and is the most common cause of cancer-related mortality [1]. Moreover, according to WHO projections, the global burden of lung cancer is expected to rise due to industrial and environmental pollution and many other factors. In 2010, a report from National Central Cancer Registry of China demonstrated that the lung cancer incidence and mortality increased to 46.08/100,000 and 37.00/100,000, respectively [2]. Non-small cell lung cancer (NSCLC) accounts for approximately 80% of all lung cancers. NCI SEER database reported that 5-year survival was 58.2% for NSCLC patients diagnosed at localized stage (18%) of all stages including localized, regional and distant from 2004–2010, whereas it was only 4.5% when diagnosed at distant stage (55%) [3]. Although many advances achieved in cancer biology as well as in diagnosis and treatment, progress in lung cancer therapy has been slow, leading to about 5% improvement in 5-year survival rates for the last 20 years [4].

Most important advances include the discovery of oncogenic driver genes and therapies specific for these genes or pathways. The common driver genes identified so far in NSCLC included the mutant activations of KRAS, EGFR, Met and BRAF [5]. In comparison with standard chemotherapy, new target therapies show a significant improved progression-free survival (PFS) and relatively less toxicity [4]. However, the median PFS of NSCLC patients with EGFR mutation was only 9.5 months on treatment with tyrosine kinase inhibitors (TKIs) [6]; The median PFS for patients with locally advanced ALK-positive lung cancer was only 7.7 months with crizotinib treatment [7]. Moreover, the overall survivals are not significantly improved for patients received target therapy as compared with chemotherapy [6]-[8]. It is obviously that overcoming refractory to target drugs can prolong treatment response. In this respect, experimental study proved that inhibition of hedhehog signaling abrogated resistance of NSCLC to TKI [9].

Novel inhibitors targeting multiple kinase sites are extensively developed, some have entered clinical trial. For example, a novel kinase inhibitor targeting both EGFR and HER2 has been evaluated in lung and breast cancer in phase I study and is expected to bring improved response [10]. Meanwhile, combination of target drugs with traditional chemotherapeutics had also been deployed, such as the conjunction of MEK inhibitors with anti-cancer drug docetaxel in previously treated patients with advanced lung cancer. Combined therapy revealed better response rate and prolonged PFS than chemotherapy alone in a phase II randomized trial [11].

In translational research, scientists performed comprehensive molecular studies to search for new genes in lung cancer. Whole-genome and transcriptome sequencing of tumor and adjacent normal tissue samples from NSCLC had identified novel alterations in genes involved in chromatin modification and DNA repair pathways, novel metabolic enzymes, as well as aberration of cell fate determination factor DACH1 [12]. Further experimental study confirmed tumor suppresser function of DACH1 in lung and other cancers [13],[14]. Most recent study to molecularly profile 230 resected adenocarcinomas revealed recurrent aberrations in multiple known pathways and previously unknown mutations of RIT1 and NF1 as new driver genes [15], suggesting the complexity of NSCLC. Recently, novel genes or pathways involved in tumor initiation and progression have been identified. For instance, metastasis associated lung adenocarcinoma transcript 1 (MALAT1), a long non-coding RNA (lncRNA), has been proven to function as a key regulator of brain metastasis in NSCLC. By inhibiting epithelial-mesenchymal transition (EMT), silencing MALAT1 reduced lung cancer cell invasion and metastasis. Clinically, protein abundance of MALAT1 correlated tightly with the poor prognosis of patients [16]. Although many risk factors have been connected tightly with the initiation and progression of NSCLC, currently there is no satisfactory strategy that can effectively prevent the cancer progression. Since extensive invasion and remote metastases severely prevent the option of surgical intervention and are key causes for lung cancer death, how to block those processes is always a critical topic for basic research and clinic management. Therapeutic refractory lung cancer cells frequently reveal EMT phenotype and signaling to block EMT has been shown to enhance chemotherapy sensitivity [17],[18]. Taken together, EMT, metastases and drug resistance are intertwined in NSCLC, which lead to aggravation and poor prognosis. Therefore, more attention should be placed on seeking potential pathways and new targets to improve treatment outcome of NSCLC.

## The role of EMT and CSCs in Metastasis of NSCLC

**Figure 1:**
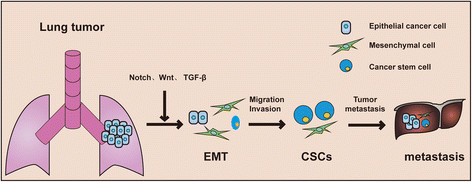
**The signaling pathways in lung cancer.** Oncogene activation or loss of tumor suppressor initiates lung tumor formation. The Notch, Wnt and TGF-β signaling pathway are involved in the induction of EMT, CSCs and metastasis of NSCLC.

EMT is a highly coordinated process, in which epithelial cells lose polarity together with cell-cell adhesion and acquire properties of mesenchymal cells. Key transcription factors driving EMT include the Snail homologues (Snail1, Snail2/Slug and Snail3), twist, zinc finger E-box-binding homeobox 1 (ZEB1) and ZEB2, and TCF3/E47/E12 [19],[20]. Multiple signaling pathways, such as transforming growth factor β (TGF-β), bone morphogenetic protein (BMP), Wnt-β-catenin, Notch, Hedgehog, and receptor tyrosine kinases participate in this transition [21] (Figure 1). In vitro studies have proved that TGF-β, integrin and JAK/STAT3 signals caused EMT in lung adenocarcinoma cells [22]-[24]. The ability to invade and disseminate of epithelial cells strongly relies on a dramatic remodeling of the cytoskeleton, leading to the invasive properties in promoting tumor metastasis accompanied with the acquisition of mesenchymal phenotype [25]. E-cadherin, the cadherin superfamily of Ca^2+^-dependent adhesion molecules, plays a key role in the regulation of cell-cell interactions [26]. Epigenetically silenced E-cadherin expression was observed in advanced NSCLC and restoration of E-cadherin expression strongly decreased the invasion/migration of tumor cells [27]. In contrast, the up-regulation of N-cadherin expression is linked to the metastasis of NSCLC, whereas inhibition of N-cadherin was demonstrated to reduce the proliferation and invasion of NSCLC [28]. Additionally, the cell-cell communication determines cell signaling, cell-cell interaction and cell polarity. For example, the intercellular cell-adhesion molecule-1 (ICAM-1), a transmembrane molecule and a distinguished member of the immunoglobulin superfamily of proteins, was involved in tumor invasion and EMT, and was also correlated with tumor differentiation grade and survival of NSCLC patient [29].

Via inducing translation suppression and mRNA degradation, micro-RNAs serve as key administrators of post-transcriptional regulation and also participate in EMT [30]. More studies have proved that post-transcriptional regulatory network, such as miR-200 family, miR-101, miR-506, and several lncRNAs regulated EMT [31]. Hypoxia is one of the fundamental biological phenomena that is intricately associated with the development and aggressiveness of a variety of solid tumors. Multiple signaling pathways such as NF-κB, PI3K/Akt/mTOR, Notch, Wnt/β-catenin, and Hedgehog as well as hypoxia-induced microRNAs (miRNAs) have been recognized as important regulators of EMT in hypoxia environment, promoting tumor progression [32],[33].

The cancer stem cell (CSC) theory proposed that the malignant phenotype was sustained by a subset of cells characterized by the capacity for self-renewal and differentiation. It is believed that CSCs are responsible for propagating growth, distant metastasis and recurrence [34]. Those cells also enriched the expression of pluripotency genes and epithelial-mesenchymal transition transcription factors, along with reduced intercellular adhesion protein expression. However, the precise origin of lung CSCs is still not clear [35]. Potential markers for identifying lung cancer stem cells include CD133, CD44, ALDH, CD166 and BMI. Analyses in multiple established and patient-derived lung cancer cells well demonstrated that subpopulation of ALDH(hi)CD44(hi) cells showed self-renewal, clonogenicity, highly tumorigenic, high invasion capacities, and resistance to chemotherapy [36]. Embryonic stem cell pathways such as Notch, Hedgehog and WNT were found active in lung cancers stem cells. Majority of human lung adenocarcinoma samples demonstrated expression of SPC, clara cell secretory protein (CCSP) and OCT4, characteristics of bronchioalveolar stem cell(BASC) [37],[38]. Experiment demonstrated that in mouse and human airway basal stem cells (ABSCs), activation of the Notch pathway was required for ROS to stimulate ABSC self-renewal [39].

Altogether, EMT is frequently associated with stemness property and therapeutic resistance, which contributes to aggressive tumor growth, invasion and metastasis, and is also recognized as the cause of tumor recurrence. In one hand, the expressions of embryo stem cell related genes are enriched on cells under EMT [38]. At the other hand, knockdown of stem cell factor, Oct4/Nanog, suppressed the expression of EMT gene Slug and reversed the EMT process, followed by reduced tumorigenic and metastatic ability. Importantly, survival time for mice bearing transplanted tumor double knockdown of Oct4/Nanog was also greatly improved [40]. Moreover, the immunohistochemical stain demonstrated expressions of Oct4, Nanog and Slug were associated in high-grade human lung adnecarcinoma tissues and the patients with triple positivity of Oct4/Nanog/Slug indicated a worse prognosis [40].

## The Notch signaling pathway in NSCLC

**Figure 2:**
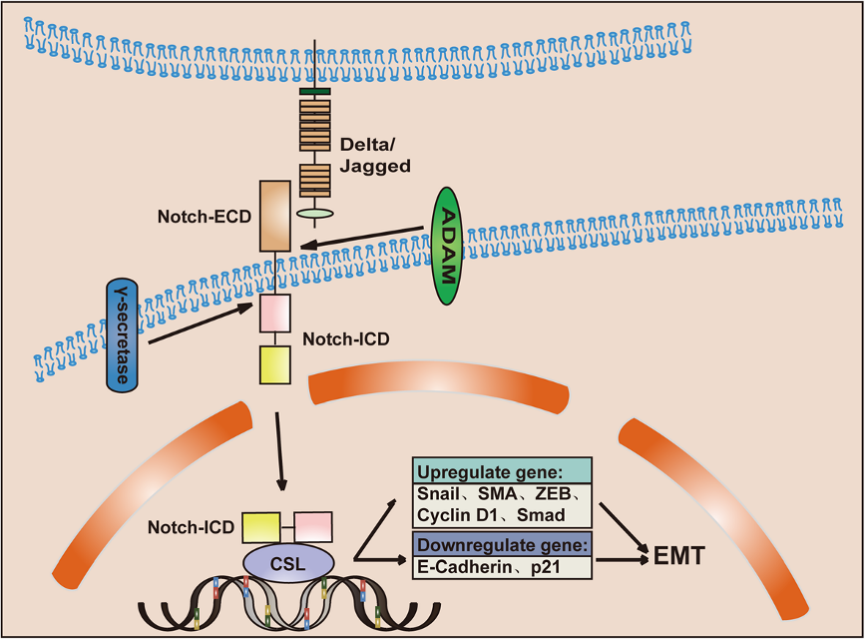
**Schematic representation of Notch signaling related to EMT.** Upon ligand binding, Notch undergoes two proteolytic cleavages by ADAM and γ-secretase complex, leading to the release of Notch-ICD and its translocating to the nucleus. In the nucleus Notch-ICD interacts with CSL and activates numerous downstream target genes: upregulation of snail, slug, zeb1, cyclinD1 and Smad; downregultion of E-cadherin, p21, etc. to induce EMT. (Notch-ECD, the extracellular domain of Notch; Notch-ICD, the intracellular domain of Notch; ADAM, a disintegrin and metalloproteinase; CSL, CBF-1/suppressor of hairless/Lag1).

The Notch signaling is activated by ligand binding to receptor to initiate an intercellular communication system [41]. There are five ligands, namely Delta-like 1, Delta-like 3, Delta-like 4, Jagged-1 and Jagged-2, and four receptor members (Notch 1 ~ 4). Ligand binding induces conformational change in Notch, leading to the exposure of S2 site for sequentially cleavage by a member of the A Disintegrin And Metalloproteinase family of proteases and the gamma secretase complex to liberates the Notch Intracellular Domain (ICD), then ICD translocates to the nucleus [42]. In the nucleus, ICD binds with the transcription factor CBF-1/suppressor of hairless/Lag1 (CSL) and modulates gene expression (Figure 2). Without ICD, CBF-1 protein binds to the consensus DNA sequence in association with SMART complex, acting as a transcriptional repressor. Interaction of CBF-1 with ICD displaces the corepressor SMART/HDACs complex, allowing for the transcriptional activation of target genes, primarily involving in two families of helix-loop-helix transcription factors: Hes (Hairy enhance of split) and Hey (Hairy/enhancer of spit related with YRPW motif) targeting genes. Other known target genes include cyclinD1, c-myc, p21, p27, the nuclear factor-kappa B (NF-κB), Survivin, Slug and Nanog [43].

Overwhelming evidence has proven that Notch signaling is crucial for the development and homeostasis of most tissues. Notch signaling has been known to participate in cell proliferation, differentiation and apoptosis. Earlier studies discovered that deregulated Notch signaling leads to various diseases, such as T cell leukemia and breast cancer [44],[45]. Notch1 mediates hypoxia-induced proliferation, invasion and chemoresistance [46]. Hypoxia also induced CD133 expression, which has been used as a stem cell biomarker of stem-like cells [47]. Subsequent study demonstrated that Notch signaling drived stemness and tumorigenicity of esophageal adenocarcinoma [48]. However, the effects of Notch signaling pathway on controlling cell fates are cell- and tissue-type dependent. Notch activation in bladder cancer cells suppresses proliferation by reducing the phosphorylation of ERK1 and ERK2 (ERK1/2). In support of this, genetic inactivation of Notch signaling in mouse models leads to Erk1/2 phosphorylation, resulting in tumorigenesis in the urinary tract [49].

The involvement of Notch on lung cancer was experimentally proved in transgenic mouse model by the alveolar epithelium specific expression of activated Notch [50]. The mice developed alveolar hyperplasias as early as 7 days’ induction of Notch and the lesions progressed to pulmonary adenomas after 8 months’ induction. Molecular analyses revealed that Notch1 activation leads to dysregulated expansion of type II lung epithelial cells. When crossed with mice conditionally overexpressing MYC in the alveolar epithelium, mice developed adenocarcinomas. Those pathological processes mimic progression of human adenocarcinoma. Using an autochthonous model of lung adenocarcinoma with concomitant expression of oncogenic Kras and deletion of Notch1, Dr. Kissil’s group found that Notch1 function was required for tumor initiation via suppression of p53-mediated apoptosis through the regulation of p53 stability [51]. Molecular analyses defined subpopulation of CD24 (+) ITGB4 (+) Notch (hi) cells were capable of propagating tumor growth in both clonogenic and an orthotopic serial transplantation assays. While all four Notch receptors represented tumor-propagating cells, Notch3 plays a nonredundant role in Kras models and in human NSCLC [52]. Interestingly, in K-Ras mouse model, inhibition of Notch strongly inhibits adenocarcinoma formation but promotes squamous hyperplasia in the alveoli. In contrast, activation of Notch leads to widespread Sox2 (+), Sox9 (+), and CC10 (+) papillary adenocarcinomas throughout the bronchioles [53]. Those researches supported a strong and direct role of Notch signaling in NSCLC. Other studies had shown that under hypoxic conditions, Notch1 stimulated NSCLC tumor growth through direct upregulation of IGF1-R [54] and survivin [55], both of which enhanced cell proliferation and survival.

In clinic study, activation of Notch signaling by either Notch1 upregulation or Numb downregulation was observed in 30% of primary human NSCLCs [56]. Notch3 also appears to be a key player in NSCLC, with overexpressed in 40% of NSCLC tumors [57]. In experimental study, either dominant-negative Notch3 receptor [58] or MRK-003, a gamma-secretase inhibitor [59], antagonized Notch3 signaling, attenuated cell growth, and induced apoptosis of lung cancer cell lines. In depth molecular analyses demonstrated crosstalk between the Notch3 and the EGFR pathway resulted in the inhibition of apoptosis through expression of anti-apoptotic protein BIM [60]. For precise target therapy, the specific region within the Notch3 extracellular domain with EGF receptor-like repeats was identified and that might be responsible for the distinct effects of Notch3 versus those of Notch1. Functional study showed peptides from these regions induced apoptosis in NSCLC [61].

Clinically oriented studies have highlighted that Notch signaling impacts survival in lung cancer patients. A recent study by Donnem *et al*. assessed the prognostic impact of Notch ligands and receptors in NSCLC and found that high Notch1 expression was statistically significantly associated with poor outcomes in lung adenocarcinoma [62]. Elevated levels of Notch-1, 3 and their ligand Jagged 1–4 expressions were found to be associated with tumor progression and predicted poor prognosis in NSCLC, suggesting a promising biomarker for NSCLC [62],[63]. Delta-like ligand 4 and Notch-1 are independent prognostic factors in NSCLC, but show diverse impacts in squamous cell carcinoma and adenocarcinoma. In addition, Jagged-1 was found to be highly expressed in metastatic NSCLC compared to localized NSCLC [63]. The coexpression of Notch-1/VEGF-A has a major impact on survival, suggesting Notch regulated angiogenesis is involved in the metastasis and determines the prognosis of NSCLC.

## Mechanism of Notch signaling in regulating EMT

Notch signaling pathway participates in an elaborate gene program and protein network for the establishment of motile and invasive mesenchymal phenotypes from polarized epithelial properties, such as cell-cell connections and cell polarity [64]. It was first revealed that Notch activity induced EMT was required for cardiac valve and cushion formation during heart development, involving down-regulation of epithelial markers (E-cadherin) and up-regulation of mesenchymal markers (snail) [65],[66]. Meanwhile, inactivation of Notch signaling by a γ-secretase inhibitor could reverse the EMT process [67]. Interestingly, over-expression of Jagged1 could trigger repression of E-cadherin in human kidney epithelial cells, suggesting Jagged-1 is a necessary element of Notch signaling for promoting EMT [68]. In EMT processes, Notch cross-talks with several transcription and growth factors relevant to EMT, including Snail, Slug, TGF-β, FGF, and PDGF [21],[69]. Key targets in Notch signaling are discussed below.

### Notch, Snail and E-cadeherin

Crystallization of Snail illustrates that it contains a conserved carboxy-terminal region with 4-to 6-C_2_H_2_-zinc fingers. By attaching through finger structures to E-box motifs (5′-CANNTG-3′) in target promoters, such as the E-cadherin gene (*CDH1*) promoter, Snail1 and Snail2 (Slug) act as transcriptional repressors [70]. The reduction of E-cadherin expression reduces the cell-cell junction and leads to destabilization of the epithelial structure, which is an initial step in EMT. Experiments proved that over-expression of Notch-ICD alone could increase snail expression and resulted in the loss of E-cadherin. On the contrary, inactivation of Notch attenuated the down-regulation of E-cadherin expression and decreased Snail expression, suggesting a direct role for Notch signaling via Snail in the induction of EMT [71]. By recruitment of hypoxia-inducible factors, HIF-1α and HIF-2α, Notch signaling pathway could also increase Snail expression to promote EMT through a sophisticated framework [72]. Interestingly, the inhibition of COX-2 could reverse the ability of Notch1 to induce EMT, suggesting that Notch is dependent on COX2 to mediate pro-metastasis via Snail/E-cadherin in EMT [73]. Recent report from Dr. Pine’s group indicates that Notch1 could regulate Sox9 expression through directly interaction with its promoter binding site to induce cell invasion and repress E-cadherin [74]. It has also been demonstrated that Slug serves as an activator in NSCLC cell lines during the induction of EMT. Furthermore, inhibition of Notch signaling led to partial reversal of EMT by decreased expressions of Snail, Slug, ZEB1, vimentin, and nuclear factor-kappaB, showing slug was essential for Notch-mediated EMT [75]. These findings linked Notch pathway and Snail/Slug tightly, making them attractive targets for new therapeutics.

### Notch and ZEB

ZEB family (ZEB1 and ZEB2), another group of transcription factor, contributes to tumorigenicity as well as EMT in NSCLC [76],[77]. ZEB1 suppresses the expression of cell polarity factors, in particular of Lgl2, which is critical for the epithelial phenotype and that its loss is involved in metastasis [78]. Correspondingly, inhibition of ZEB1 reverses EMT and chemo-resistance in docetaxel-refract human lung adenocarcinoma cell line [79]. Immunohistochemical analyses of clinical sample revealed that ZEB1 and twist are more commonly expressed in metastatic than primary lung tumors and show inverse associations with claudins [80]. Subpopulation of metastasis-prone mouse lung adenocarcinoma cells expresses high level of Notch and Notch ligands, but decreased miR-200. Reduction in Notch signaling resulted in reduced proliferation, increased apoptotic susceptibility, and decreased tumorsphere formation [81]. It is concluded that interaction of Notch-miR-200-ZEB1 loop governed EMT and metastatic capacity. This novel Notch/miR-200-dependent pathway, which mediates lung adenocarcinoma metastasis in mice, may provide new target for the treatment of human epithelial tumors.

### Notch and TGF-β

The transforming growth factor beta (TGF-β), a ubiquitous cytokine with profound growth inhibitory effects on epithelial and other tissues, orchestrates an intricate signaling network that is crucial to determine cell fate, differentiation, proliferation and motile, leading to not only tumour suppression but also EMT [82]. The TGF-β superfamily consists of the TGF-β family (TGF-β1, TGF-β2 and TGF-β3), NODAL, activins, and bone morphogenetic proteins (BMPs). Initiated by ligand-receptor binding, TGF-β mediates the interaction of SMAD to the promoters of Snail, contributing to the development of EMT in NSCLC [83],[84]. TGF-β induced EMT could be blocked by knock down of Hey-1 or jagged-1 and by pharmacological inactivation of Notch, indicating the key role of Notch signaling in TGF-beta-induced EMT [85]. Meanwhile, inhibition of Notch signaling could significantly inhibit TGF-β-induced expression of SMA, suggesting Notch induced EMT through a TGF-β-Smad3 pathway that activates SMA gene transcription [86]. However, in cultured limbal progenitor cells, Notch prevents TGF-β-assisted EMT through the induction of Smad7 [87]. While in breast cancer, Notch pathway inhibits TGF-β signaling through HEYL-mediated crosstalk, promoting initiation of breast cancer [88]. Collectively, these studies indicate that the cross talk between Notch signaling and TGF-β play a profound role in EMT.

### Notch and Cyclin D1

Hyper-activation of cyclins, especially the G1/S administrator cyclin D1 may favor tumor development by inducing unscheduled cell division in progenitor cells [89]. The abundance of cyclin D1 has a significant relationship with neoplastic progression of NSCLC [90],[91]. In epithelial ovary cancer cells, stem-like CD24- cells or spheroids highly expressed cyclin D1, Bmi-1, and vimentin with reduced expression of E-cadherin, while non stem-like CD24+ or parental cells showed the opposite expression. Furthermore, cyclin D1-targeted small interfering RNA resulted in decreased vimentin expression in spheroids, accompanying with reduced cell viability and migration [92]. In cervical squamous carcinoma, most invasive tumour cells expressed cyclin D1 and showed a reduction in E-cadherin and beta-catenin staining [93]. Epidermoid A431 cells expressing SIP1 along with exogenous cyclin D1 were highly invasive, indicating that SIP1-regulated invasion is independent of attenuation of G1/S progression [94]. Conversely, knock down Notch-3 reduced cyclin D1 with reduced proliferation and enhanced apoptosis [95]. Given the importance of Notch and cyclin D1 in tumorigenesis, it is not surprising that Notch pathway would participate in regulation of cyclin D1-mediated EMT. Activation of Notch signaling during neural induction in the ES cells led to significantly enhanced cell proliferation, accompanied by Notch-mediated activation of cyclin D1 expression. A reduction of cyclin-D1-expressing cells was observed in the developing CNS of Notch signaling-deficient mouse embryos. Moreover, expression of a dominant negative form of cyclin D1 in the ES cells abrogated the Notch-induced proliferative ability, and, conversely, a constitutively active form of cyclin D1 mimicked the effect of Notch on cell proliferation [96]. Gifitinib-acquired resistant lung cancer cells displayed EMT phenotype. Molecular analyses confirmed that activation of Notch-1 and its target genes, which induced expression of mesenchymal marker vimentin, snail and hes-1, and decreased epithelial marker E-cadherin. The elevated expression of Notch-1 could directly suppress p21 Waf1/Cipl accompanied with the increasing of cyclin D1, giving rise to the EMT phenotype. Meanwhile, knockdown of Notch-1 decreased cyclinD1 expression and reversed EMT. These findings suggest that the aggressive behavior caused by cyclinD1 might be driven by the mis-expression of Notch pathway in NSCLC, which finally down regulates p21 Waf1/Cipl and promotes the EMT phenotype [71].

## Targeting Notch signaling in NSCLC

*In vivo* evidence that Notch inhibitor is a potential therapeutic agent came from Kras(G12V)-driven NSCLCs mice model. Pharmacologic treatment of mice carrying autochthonous NSCLCs with a γ-secretase inhibitor (GSI) blocked cancer growth. Correspondingly, molecular analysis of treated cancer tissues demonstrated reduced HES1 levels and phosphorylated ERK [97]. Currently, several classes of investigational Notch inhibitors have been developed. These include monoclonal antibodies against Notch receptors or ligands, decoys to soluble forms of the extracellular domain of Notch receptor or Notch ligands, blocking peptides, and gamma-secretase inhibitors (GSIs) or natural compounds [98].

At present, GSIs are the most extensively explored. RO4929097, a small-molecule inhibitor of GSI with high oral bioavailability and is a potent and selective inhibitor of gamma-secretase, has been tested in phase I study in refractory metastatic or locally advanced solid tumors [99], and phase II studies for metastatic melanoma [100], metastatic colorectal cancer [101] and metastatic pancreatic adenocarcinoma [102]. Another GSIs, PF-03084014, was also evaluated on phase I in advanced solid tumor [103]. In preclinical study, MRK-003 was evaluated in triple negative breast cancer cells by MRK-003 alone and in combination with paclitaxel. Immunohistochemical staining for activated NOTCH1 and HES4 expression could be molecular biomarkers, identifying solid tumors that are likely to respond to GSI-based therapies [104]. Preclinical study of MRK-003 in pancreatic cancer [105] and in multiple myeloma and non-Hodgkin’s lymphoma exhibited promising activity [106]. Treatment with GSIs MK-0752 in breast cancer cell lines reduced stem cell subpopulation *in vitro* and in human tissues from clinical trial [107]. Clinical benefit of MK-0752 in adult patients with advanced solid tumors was observed with well tolerated toxicity in Phase I study, therefore promoting to combinational trials [108].

Inhibitors of Notch signaling can be used not only as direct anti-cancer agents but also as a sensitizer to current therapy. Platinum-based chemotherapy is the first-line treatment for NSCLC, but recurrence occurs in most patients. Experimental study found that treatment of NSCLC cell line H460 and H661 enriched CD133 (+) cells and upregulated ABCG2 and ABCB1 expression, which conferred the cross-resistance to doxorubicin and paclitaxel. Detailed molecular analysis found that the enrichment of CD133 (+) cells by cisplatin depended on Notch signaling. Moreover, pretreatment with the γ-secretase inhibitor or Notch1 short hairpin RNAs (shRNA) remarkably increased the sensitivity to doxorubicin and paclitaxel. Importantly, similar phenomena were observed both in engrafted tumors derived from transplanted animal model and the relapsed tumors of patients who had received cisplatin treatment [109]. Gamma-secretase inhibitor DAPT alone slightly inhibited the proliferation and exhibited little effect on the cell cycle, but enhanced the inhibitory effects of Cisplatin in a combinational study with GSI. Interestingly, this effect was especially significant in CD133 (+) cells, suggesting that Notch pathway blockade may be a useful CSC-targeted therapy in lung cancer [110]. In complementary, Dr. Carbone’s group found that treatment of EGFR-mutated lung cancer cell lines with erlotinib enriched the ALDH^+^ stem-like cells with stem-like cell potential through EGFR-dependent activation of Notch3. Moreover, γ secretase inhibitor could reverse this phenotype. At molecular level, physical association between the Notch3 and EGFR receptors leads to tyrosine phosphorylation of Notch3. This study could explain the unflavored survival observed in some studies of erlotinib treatment at early-stage disease, and imply that specific dual targeting might overcome adverse effect of TKIs [111].

γ-Secretase inhibitor administration after radiation had the greatest growth inhibition of lung cancer i*n vitro* and *in vivo*. Mechanically, enhanced apoptosis of lung cancer cell lines in combination group were through regulation of MAPK and Bcl-2 family proteins. Furthermore, radiation-induced activation of Notch was blocked by GSI administration, suggesting that treatment with GSI could prevent Notch-induced radiation resistance [112]. Together, above studies provided compelling evidence for exploiting Notch inhibitors in clinic trial. In most cases, the application of GSI is limited because of gut toxicity and goblet cell hyperplasia. Therefore, the best dose and schedule need to be optimized.

Other receptor-specific approaches like siRNA directed against Notch might be useful in reducing the tumorigenicity and invasive of NSCLC. For instance, nanoparticle (NP) technology has been applied to deliver specific siRNA to knockdown Notch1 to arrest tumor growth and reverse EMT by the up-regulation of miR-200a and down-regulation of the transcription factors ZEB1, ZEB2, Snail and Slug [113]. Furthermore, monoclonal antibody against Notch can also reduce the invasion of NSCLC [114],[115]. The complex of γ-secretase is a transmembrane protease that catalyses the cleavage of a set of membrane proteins and is comprised of four subunits encoded by four genes, including PSEN1, PSENEN, NCSTN and APH1. Targeting NCSTN using specific mAbs may represent a novel mode of treatment for invasive triple-negative breast cancer, for which there are few targeted therapeutic options. It is suggested that measuring NCSTN in patient samples may serve as a molecular marker for anti-NCSTN therapy in the clinic [116].

>Some natural agents such as curcumin, 3,3′-diindolylmethane (DIM), resveratrol, and epigallocatechin-3-gallate (EGCG) have been reported to be effective on targeting Notch signaling, suggesting they can also be promising alternative strategies for NSCLC chemotherapy [117],[118].

## Conclusion

As a well known driving force of cell invasion, migration and metastasis, EMT has been shown to induce resistance of cancer cells to conventional chemotherapy and radiotherapy. Notch signaling not only activates cell proliferation, antagonize apoptosis but also cross-talks with several transcriptional factors to promote EMT, leading to enhanced motility *in vitro* and invasion and metastasis *in vivo*. Theoretically, combination of chemotherapy or radiotherapy with Notch inhibitors might acquire synergistic effect and improve chemotherapy response. Although promising results have been noticed in some patients with Notch inhibitors in clinical trials, stratification biomarkers to identify patients who are most likely benefit from GSIs treatment are required for a successful development of this class of drugs.

## Authors’ original submitted files for images

Below are the links to the authors’ original submitted files for images. Authors’ original file for figure 1 Authors’ original file for figure 2
