# Supplementary figures and images for: Notch signaling and EMT in non-small cell lung cancer: biological significance and therapeutic application
Source: J Hematol Oncol. 2014 Dec 5;7:87. doi: 10.1186/s13045-014-0087-z (PMC4267749; doi:10.1186/s13045-014-0087-z)

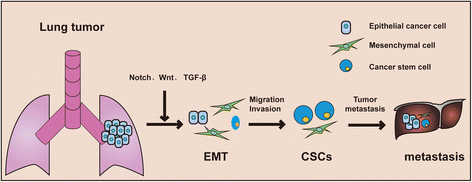

Supplement: Supplementary file 1 — Authors’ original file for figure 1 [file 13045_2014_87_MOESM1_ESM.gif]

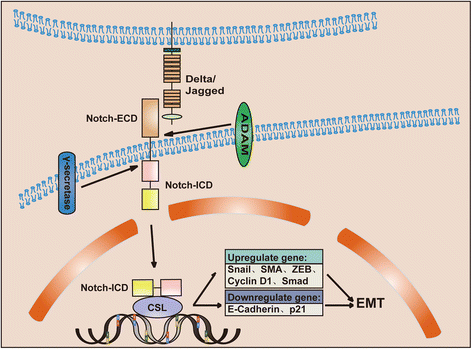

Supplement: Supplementary file 2 — Authors’ original file for figure 2 [file 13045_2014_87_MOESM2_ESM.gif]
